# The Association between the Five-factor Model of Personality and Problem Gambling: a Meta-analysis

**DOI:** 10.1007/s10899-022-10119-5

**Published:** 2022-05-23

**Authors:** Francine W. H. Dudfield, John M. Malouff, Jai Meynadier

**Affiliations:** University of New England Psychology, Armidale, NSW 2351 Australia

## Abstract

This meta-analysis examined the associations between five-factor personality model traits and problem gambling. To be eligible for inclusion in the meta-analysis, studies had to provide effect size data that quantified the magnitude of the association between all five personality traits and problem gambling. Studies also had to use psychometrically sound measures. The meta-analysis included 20 separate samples from 19 studies and 32,222 total participants. The results showed that problem gambling was significantly correlated with the five-factor model of personality. The strongest personality correlate of problem gambling was neuroticism *r* = .31, *p* = < 0.001, 95% *CI* [0.17, 0.44], followed by conscientiousness *r* = − .28, *p* = < 0.001, 95% *CI* [-0.38,-0.17] ), agreeableness *r* = − .22, *p* = < 0.001, 95% *CI* [-0.34, − 0.10], openness *r* = − .17, *p* = < 0.001, 95% *CI* [-0.22,-0.12], and extraversion *r* = − .11, *p* = .024, 95% *CI* [-0.20,-0.01]. These results suggest problem gamblers tend to share a common personality profile – one that could provide clues as to the most effective ways to prevent and to treat problem gambling.

## The Association between the five-factor model of personality and Problem Gambling: a Meta-analysis

Problem gambling, also termed gambling disorder and pathological gambling, is a behavioral addiction characterized by persistent gambling behavior despite significant negative consequences that can include financial hardship, legal problems, relationship and occupational dysfunction, and significant emotional distress (Blanco & Bernadi, 2014); Brunborg et al., 2016). The diagnostic criteria for gambling disorder in the Diagnostic and Statistical Manual of Mental Disorders − 5 (DSM-5; American Psychiatric Association, 2013) includes additional elements, such as restlessness or irritability when reducing or attempting to stop gambling, a preoccupation with gambling, and a tendency for gambling to occur when feeling distressed.

Problem gambling is associated with stress, depression and anxiety, feelings of shame and worthlessness (Australian Government Productivity Commission, 2010), along with suicide ideation and attempts (Gray et al., 2021; Wardle & McManus, 2021).

Problem gambling is not rare. Worldwide prevalence is estimated to range from 0.5 to 7.6% (Williams et al., 2012). A recent analysis estimated the societal costs of problem gambling in Sweden alone to be about $2 billion (Hofmarcher et al., 1921).

Researchers have considered various factors that might contribute to problem gambling pathogenesis and have suggested a multi-factorial model consisting of biopsychosocial factors (Shaffer et al., 2004). Researchers view personality as playing an influential role in the development, manifestation, severity, and maintenance of gambling disorder (Bagby et al., 2007; Mackinnon et al., 2016; Takada & Yukawa, 2019).

Personality traits are enduring characteristics that are consistent and stable across time and situation (Gregory, 2011). Personality is immensely complex. The most prominent and psychometrically supported model of personality in psychology is Costa and McCrae’s (1997) five-factor model of personality (Baranczuk, 2019). According to the five-factor model of personality, there are five broad personality domains that can describe between-person differences in human personality: openness, conscientiousness, extraversion, agreeableness, and neuroticism (McCrae & Costa, 1997). Openness is the tendency to be imaginative, curious, and have an open-mind; conscientiousness is the tendency to be well organized, goal oriented, and self-disciplined; extraversion is the tendency to be assertive, energetic, and sociable; agreeableness is the tendency to be affectionate, cooperative, helpful, and trusting; neuroticism is the tendency to feel anxious, irritable, depressed, and insecure (Mackinnon et al., 2016; Shum et al., 2013).

In studies examining the five-factor model and problem gambling, researchers have used various psychometric instruments to assess problem gambling. The South Oaks Gambling Screen (SOGS; Lesieur & Blume, 1987), which measures the severity of disordered gambling behaviors, consists of 20 items including: (1) *Did you ever gamble more than you intended to?* and (2) *When you gamble, how often do you go back another day to win back money you have lost*? A score of five or more indicates probable problem gambling. The 9-item Problem Gambling Severity Index (PGSI; Ferris & Wynne, 2001) assesses behaviors and consequences associated with problem gambling. Responses are based on the frequency of the behaviors. Items include: (1) *Have you borrowed money or sold anything to gamble?* and (2) *Has gambling caused you any health problems, including stress or anxiety*?

In these studies, researchers have used various instruments that measure the five-factor model of personality. The NEO (Costa & McCrae, 1992) set of personality inventories consists of items that measure all five traits. Other standardized psychometric instruments that measure the five-factor model of personality include the Big Five Inventory (BFI; John et al., 1991), the Ten-Item Personality Inventory (TIPI; Gosling, et al., 2003), and the International Personality Item Pool (IPIP; Goldberg et al., 2006).

Some studies have found a common personality profile of problem gamblers (Mann et al., 2017; Miller et al., 2013; Quilty et al., 2021). However, not all studies have replicated these findings; for example, some studies have found that gambling disorder is associated with high neuroticism but not with the other five-factor domains (MacLaren et al., 2015; Kaare et al., 2009; Hwang et al., 2012).

Prior meta-analyses have examined the relationship between the five-factor model of personality and various types of psychological problems, including symptoms of clinical disorders of various types (Malouff et al., 2005), smoking (Malouff et al., 2006), excessive drinking (Malouff et al., 2007), and the dramatic and emotional cluster (cluster B) of personality disorders (Samuel & Widiger, 2008). In each meta-analysis, there were significant associations between multiple five-factor traits and the psychological problem. For instance, Malouff et al. (2005) found that all of the five-factor traits except openness were related to symptoms of clinical disorders. Samuel and Widiger found significant associations between all of the five traits except openness and multiple personality disorders.

The relationship between personality and problem gambling is not yet clear. Where the overall pattern of findings among related studies is not clear, a meta-analysis can be useful, so we set out to complete a meta-analysis of the association between the five-factor model of personality and problem gambling. We focused on studies using the five-factor model because we wanted to use an empirically supported model and because studies using the model provide an opportunity to assess each five-factor trait against the others in the same sample of participants. Because there have been numerous studies of the five-factor model and problem gambling, it is clear that researchers consider the relationship important. What is missing is an aggregation of the findings in a meta-analysis.

## Aims of the meta-analysis

The purpose of the present meta-analysis was to examine the association between the five-factor model of personality and problem gambling. We hypothesized that high neuroticism, low conscientiousness, and low agreeableness would be associated with problem gambling, because these personality traits have been found to be associated with other types of addictive behavior involving alcohol, cannabis, tobacco, and Internet gaming (Dash & Slutske, 2019; Malouff et al., 2005; Müller et al., 2014).

## Method

### Eligibility criteria

Studies had to meet three criteria for inclusion in the meta-analysis: (1) The related report had to include effect sizes for the association between each of the five-factor personality traits and problem gambling, (2) the report had to state the number of participants, and (3) the study had to use psychometrically sound measures.

### Search strategy

A protocol for this meta-analysis was published in the International Prospective Register of Systematic Reviews, registration number: CRD42021237773, in February 2021. In March 2021, two researchers systematically searched the following databases: EBSCO, EBSCO Open Dissertations, Google Scholar, ProQuest, and PubMed. Keywords used were five factor or big five, and gambl*. No date or language parameters were set for the electronic search. To reduce the search results in the Proquest database from 6283 to 112, we added quotation marks to the search terms “five-factor” and “big 5.” In August 2021, we repeated the electronic database search and included a date parameter set to 2021–2022 to find any recently published studies. We also examined reference lists of included articles and emailed corresponding authors of included studies requesting unpublished data. No unpublished studies were found.

### Data extraction and coding

One researcher manually extracted data from the included studies and recorded it on an electronic spreadsheet. Data extracted to calculate the effect sizes included correlations, and independent group means and standard deviations of problem gambling and healthy control groups. Coded descriptive data included: (1) study authors and publication date, (2) number of participants, (3) mean age, (4) percentage female, (5) five-factor model of personality measure used, (6) problem gambling measure used, (7) study design (correlational or between groups), (8) evidence of validity and reliability of the measures used, and (8) sample type. Then a second researcher checked data entries, and a third researcher independently coded entries needed to calculate effect sizes. Inter-rater reliability between the first two coders and the independent coder was 93%. Consensus between coders resolved all disagreements.

### Data **analysis**

Analyses were performed using the Comprehensive Meta-Analysis software (CMA Version 3.3.070; Borenstein et al., 2014). A composite score was computed for studies reporting multiple outcomes for a single trait based on the same participants. Effect sizes were calculated using a meta-analysis analogue of Pearson’s *r*. For the 13 studies that reported means and standard deviations for groups of problem gamblers and others, Hedge’s *g* (Hedges, 1982) was calculated and then converted to *r*.

We used a random-effects model because it recognizes within-study and between-study variance and assumes that the true effect size differs among studies (Borenstein et al., 2009). To measure heterogeneity, both the *I²* statistic and Cochran’s *Q* were calculated. The *I*^2^ statistic quantifies the level of heterogeneity (Higgins et al., 2003). *I*^2^ is the proportion of variance across studies that is due to true effects rather than sampling error. The Cochran’s *Q* statistic was computed to examine whether all studies in the present meta-analysis assessed the same effect (Higgins et al., 2003).

### Quality assessment

Assessment of study quality involved evaluating the validity and reliability of all measures used in studies included in this meta-analysis.

## Results

### Study selection

**Fig. 1:**
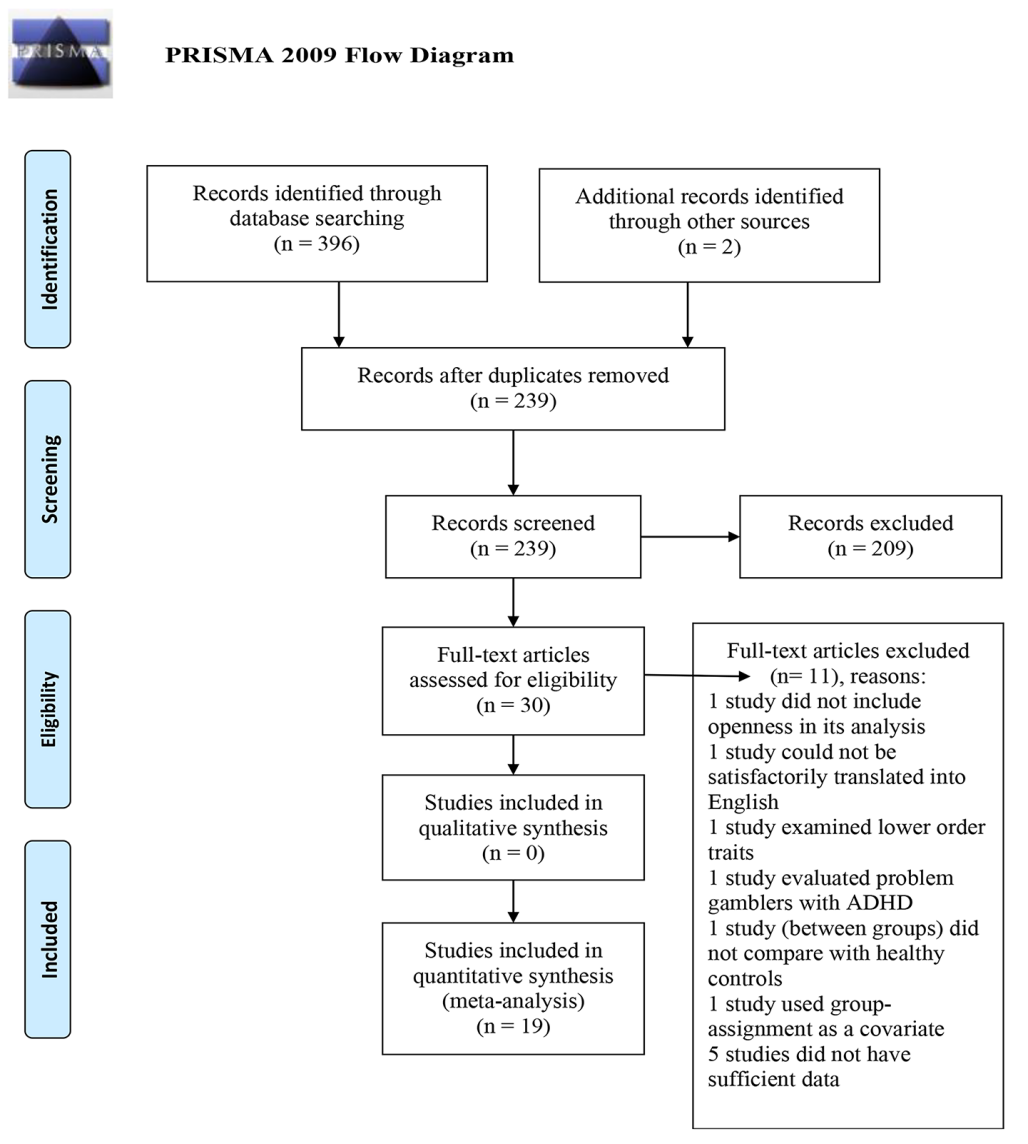
PRISMA Flow Diagram

Following the removal of 159 duplicates, 239 records were retained for screening. Of the 239 records, 20 studies seemed to meet the inclusion criteria. Out of these 20 studies, one was removed because group-assignment in a treatment study was treated as a covariate in the key reported results. Hence, 19 studies were included in the meta-analysis. One study had two independent samples, leading to a total of 20 samples to analyze. Figure 1 presents a PRISMA Flow Diagram (Moher et al., 2009) containing information about the study selection process.

### Study characteristics

Table 1 shows key study characteristics. The meta-analysis included 20 samples, with a total of 32,222 participants and produced 100 effect sizes (20 effect sizes for each of the five personality factors). The most common problem gambling scale used was the SOGS, and 12 studies used a version of the NEO to measure the five-factor model of personality. The data file is at https://rune.une.edu.au/web/handle/1959.11/31788.

**Table 1:** Descriptive information about studies included in the meta-analysis

Study	Country	*N*	% Female	Problem gambling measure	FFM of personality measure	Study design	Sample type
Bagby et al., 2007	Canada	283	52	SDA DSM-IV	NEO PI-R	*M*	Community
Brunborg et al., 2016	Norway	9111	52	PGSI	Mini-IPIP	*M*	Community
Buckle et al., 2013	Canada	212	71	SOGS	NEO FFI	*r*	Convenience
Carlotta et al., 2015	Italy	110	49	Lie Bet	BFI	*M*	Community
Cerasa et al., 2018	Italy	200	7	SOGS	NEO PI-R	*r*	In treatment for gambling
Crossman, 2007	USA	206	53	CPGI	IPIP-NEO-PI	*M*	University students
Dash et al., 2019	Australia, USA	3785	64	NODS	NEO PI-R	*r*	Australian Twin Registry
Gong & Zhu, 2019	Australia	4100	49	CPGI	HILDA Big-Five	*M*	Representative
Hwang et al., 2012	Korea	48	0	SOGS	NEO PI-R	*M*	Clinical & community
Kaare et al., 2009	Estonia	69	11	SOGS	EPIP- NEO	*M*	Clinical & community
Mann et al., 2017	Germany	113	0	SOGS	NEO FFI	*M*	Clinical & community
Miller et al., 2013	USA	354	22	SCI-PG	BFI	*r*	Frequent gamblers
Müller et al., 2014	Germany	215	0	BIG	NEO FFI	*M*	Clinical & community
Myrseth et al., 2009	Norway	156	27	SOGS	NEO FFI	*M*	Diagnosed & community
Quilty et al., 2021	Canada	134	50	CPGI, SOGS	NEO PI-R, SIFFM	*M*	Diagnosed & community
Tabri et al., 2017	Canada, USA	197	44	PGSI	TIPI	*r*	Community
Von der Heiden & Egloff, 2021	Germany	12,556	54	PGSI	36-item Big Five	*r*	Community HILDA
Whiting et al., 2019	USA	248	35	SOGS	NEO PI-R	*M*	Community
Zilberman et al., 2018	Israel	125	46	SOGS	BFI	*M*	Community problem gamblers

*Note. N =* sample size; % female = percentage of females in sample; *M* = comparison of between group means; *r* = correlation design. Abbreviations: SDA DSM-IV Structured diagnostic assessment DSM-IV; CPGI, Canadian Problem Gambling Index; NODS, The National Opinion Research Center DSM Screen for Gambling Problems; SCI-PG, structured clinical interview for pathological gambling; BIG, Berlin Inventory of Gambling Behavior; NEO PI-R, NEO Personality Inventory-Revised; Mini IPIP, International Personality Item Pool; NEO FFI, NEO Five-Factor Inventory; EPIP NEO, Estonian Personality Item Pool- NEO; and SIFFM, Structured Interview for the Five-Factor Model of Personality

### Quality Assessment

All measures used in studies included in the present meta-analysis demonstrated reliability (see Table 2). All measures used in the present meta-analysis also had evidence of validity.

**Table 2:** Reliability of measures used in the meta-analysis

Measure	Internal consistency (Cronbach’s alpha)
BIG	*a* = 0.96 (Wejbera et al., 2017)
CPGI	*a* = 0.92 (Arthur et al., 2008); *a* = 0.89 (Back et al., 2015)
Lie Bet	*α* = 0.60 (Wieczorek et al., 2021)
NODS	*a* = 0.84 (Back et al., 2015); a = 0.86 (Wulfert et al., 2005); a = 0.79 (Hodgins, 2004)
PGSI	*a* = 0.90 (Orford et al., 2010); a = 0.90 (Brunborg et al., 2016); *a* = 0.84 (Ferris & Wynne, 2001) *α* = 0.84 ((Wieczorek et al., 2021)
SCI-PG	*a* = 0.73 (Walker et al., 2006)
SDA DSM-IV	*a* = 0.92 (Stinchfield et al., 2005)
SOGS	*a* = 0.86 (gambling treatment), *a* = 0.69 (community) (Stinchfield, 2002) *a* = 0.83 (Arthur et al., 2008); *a* = 0.85 (Wulfert et al., 2005); a = 0.78 (Hodgins, 2004)
BFI	*a* = 0.72 − 0.81 (Carlotta et al., 2015); *a* = 0.73 − 0.82 (Miller et al., 2013)
EPIP NEO	*a* = 0.89 − 0.95 (Mõttus et al., 2006)
IPIP NEO PI	*a* = 0.87 − 0.94 (Sleep et al., 2021); a = 0.91 − 0.94 (Maples-Keller et al., 2019)
MINI IPIP	*a* = 0.67 − 0.78 (Brunborg et al., 2016); *a =* 0.82 − 0.87 (Sleep et al., 2021)
NEO FFI	*a =* 0.66 − 0.90 (Myrseth et al., 2009); *a* = 0.67 − 0.81 (Miller et al., 2013) *a* = 0.76 − 0.85 (Maples-Keller et al., 2019)
NEO PI-R	*a* = 0.83 − 0.90 (Mõttus et al., 2006); *a* = 0.90 − 0.93 (Maples-Keller et al., 2019)
SIFFM	*a* = 0.72 − 0.89 (Trull et al., 1998)
HILDA Big-Five	*a* = 0.74-0.81 (Losoncz, 2009)
TIPI	*a =* 0.52 − 0.70 (Ehrhart et al., 2009); *a* = 0.51 − 0.83 (Sleep et al., 2021)
36-item Big Five	*a* = 0.66 − 0.79 (Wortman et al., 2012; Lucas & Donnellan, 2009)

Studies showed concurrent validity across six of the problem gambling measures: CPGI and SOGS (*r* = .83, Stevens & Young, 2008); NODS and SOGS (*r* = .71, Wulfert et al., 2005); SCI-PG and SOGS (*r* = .78, Grant et al., 2004); PGSI and SOGS (*r* = .83, Ferris & Wynne, 2001); PGSI and DSM-IV criteria for gambling disorder (*r* = .82, Orford, 2010); and SOGS and DSM-IV criteria for gambling disorder (*r* = .66, Goodie et al., 2013; r = .72 Tang et al., 2010). Sensitivity and specificity of the Lie Bet questionnaire was 92% and 96%, respectively (Götestam et al., 2004), and Grant et al. reported 88% sensitivity and 100% specificity for the SCI-PG (2004). Wejbera et al. reported discriminant validity of the BIG (2017). Lastly, the structured diagnostic assessment demonstrated convergent validity with the SOGS (*r* = .59, Beaudoin & Cox, 1999; r = .81, Cox et al., 2004).

Studies demonstrated evidence of convergent validity across all eight five-factor model of personality measures: BFI and NEO PI-R (mean *r* = .78, Rammstedt & John, 2007); EPIP NEO and NEO PI-R (*r* = .73, Kaare et al., 2009); IPIP NEO PI and NEO FFI (mean *r* = .80, Maples-Keller et al., 2019); NEO PI-R and SIFFM (*r* = .65 − .84, Trull et al., 1998); SDA DSM-IV and SOGS (*r* = .90, Stinchfield et al., 2005); and TIPI and BFI (*r* = .65 − .87, Gosling et al., 2003). Further, the Saucier (1994) scale showed convergent validity with the 36-item Big Five and Goldberg’s (1992) five-factor model of personality adjectives (1994). The MINI IPIP exhibited criterion validity (Baldasaro et al., 2013), and a factor analysis supports construct validity of the HILDA Big-Five measure (Losoncz, 2009).

### Main results

Neuroticism had a moderate positive relationship with problem gambling *r* = .31, *p* = < 0.001, 95% CI [0.16, 0.44]. Conscientiousness showed a small negative correlation with problem gambling, *r* = − .28, *p* = < 0.001, 95% CI [-0.37,-0.17]. Similarly, agreeableness (*r* = − .22, *p* = < 0.001, 95% CI [-0.34, − 0.10]), openness (*r* = − .17, *p* = < 0.001, 95% CI [-0.22,-0.12]), and extraversion (*r* = − .10, *p* = .047, 95% CI[-0.19,-0.00]) all showed small negative correlations with problem gambling. Neuroticism and conscientiousness accounted for 9.6% and 7.8% of the variance in problem gambling scores, respectively. Agreeableness, openness, and extraversion explained 4.8%, 2.9%, and 1.2% of variance in problem gambling scores, respectively.

**Fig. 2:**
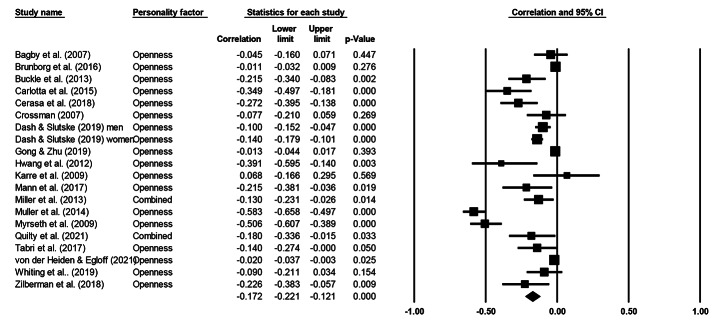
Meta-analysis on the association between openness and problem gambling

**Fig. 3:**
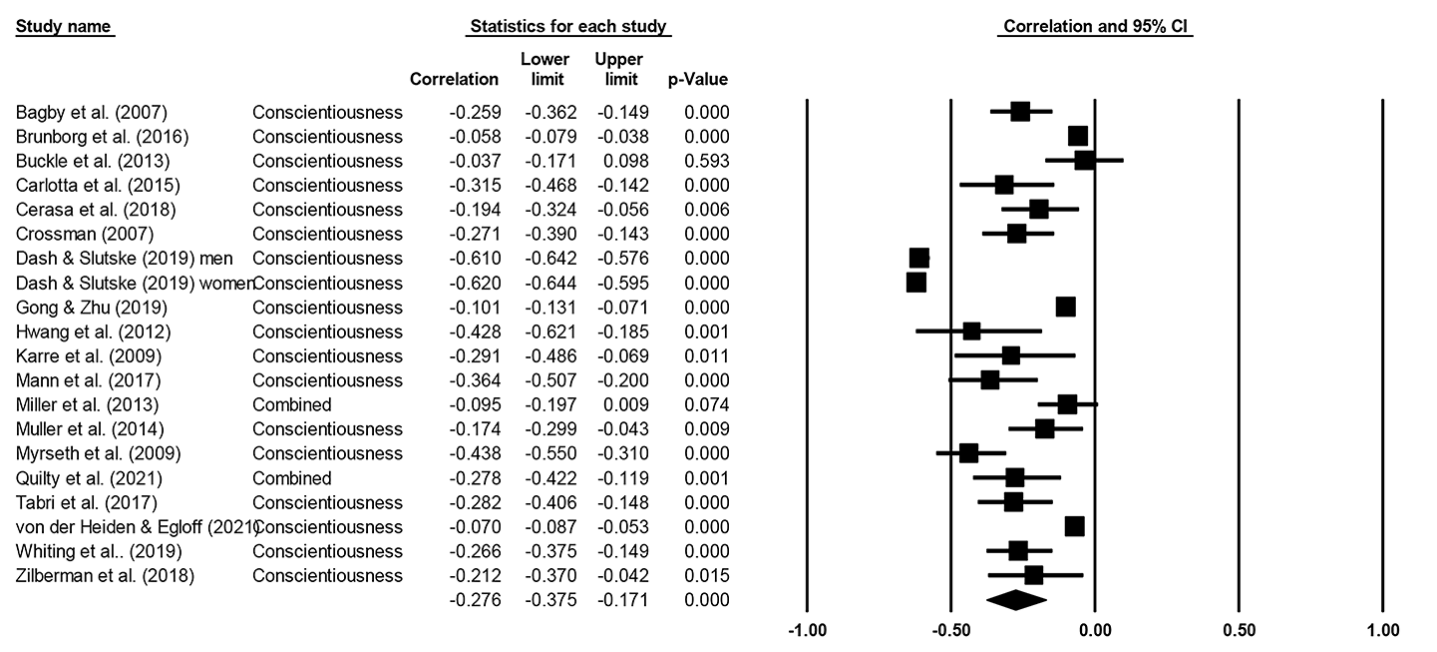
Meta-analysis on the association between conscientiousness and problem gambling

**Fig. 4:**
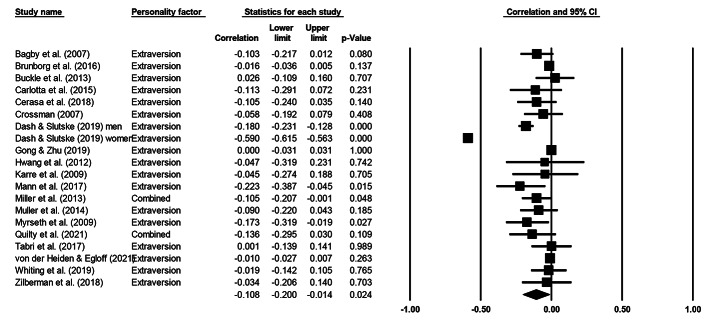
Meta-analysis on the association between extraversion and problem gambling

**Fig. 5:**
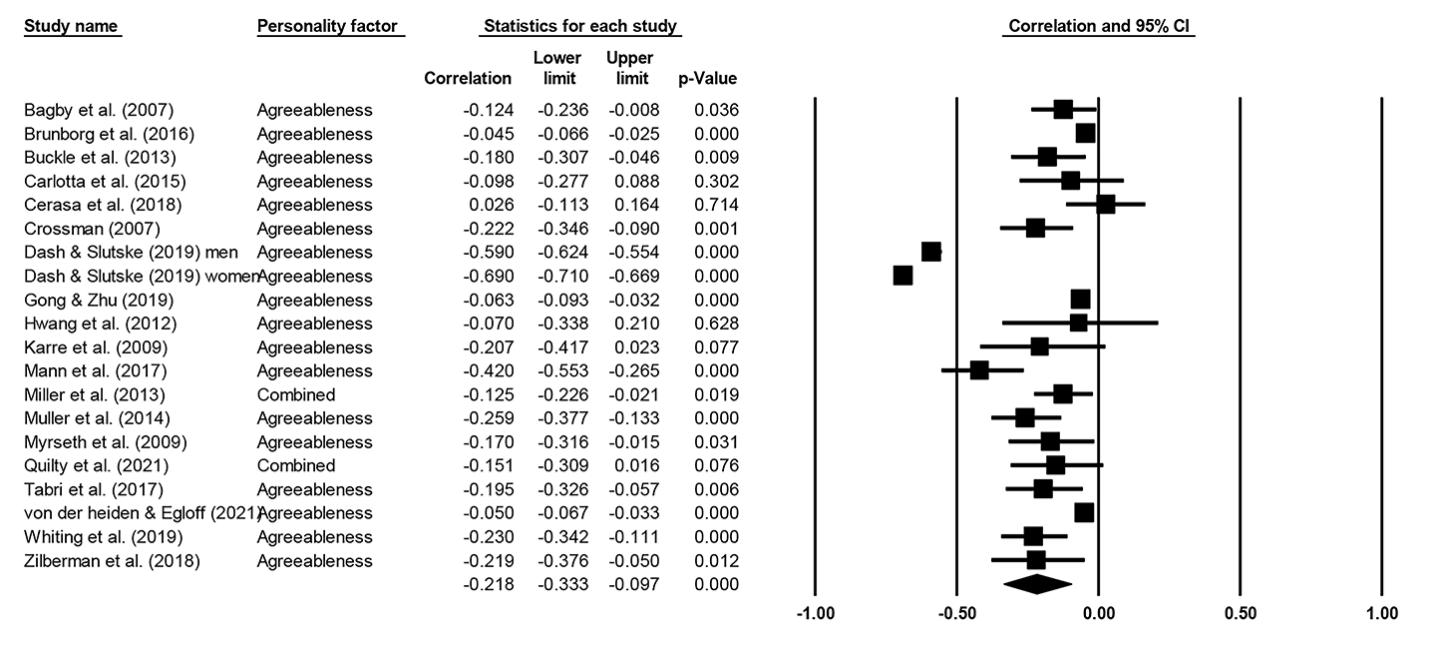
Meta-analysis on the association between agreeableness and problem gambling

**Fig. 6:**
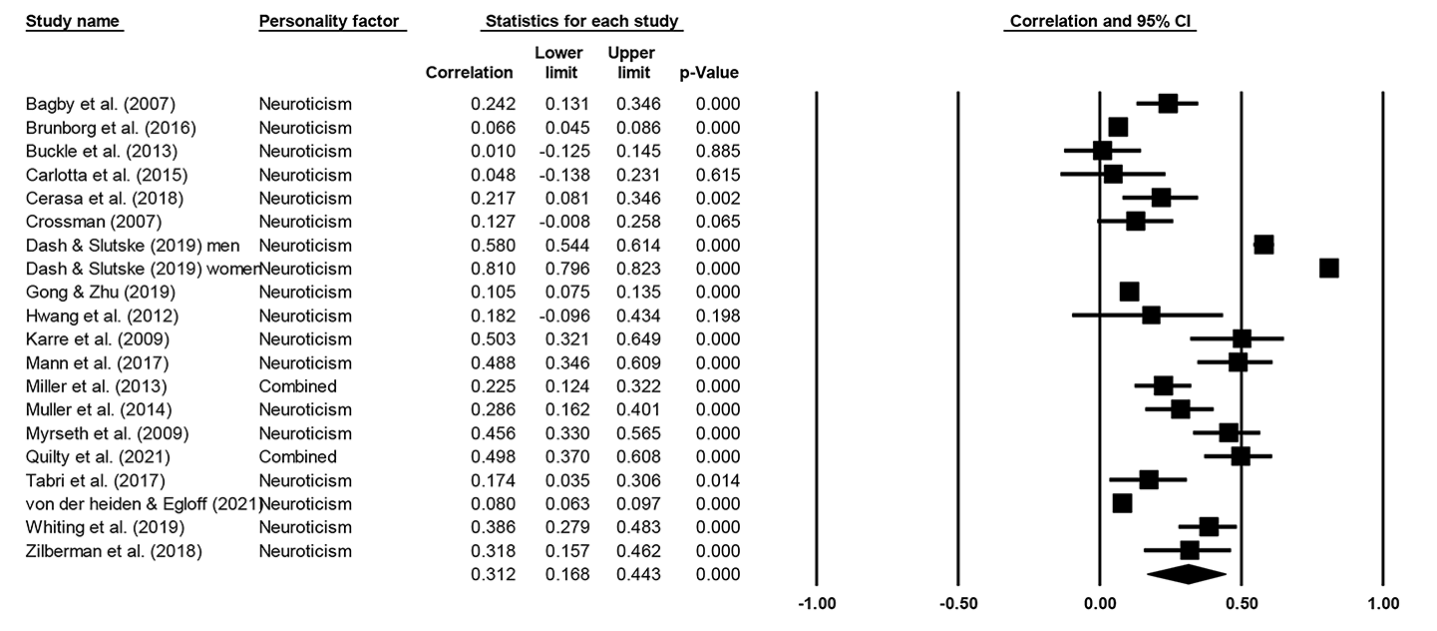
Meta-analysis on the association between neuroticism and problem gambling

Cochran’s *Q* statistic was significant across all five personality factors, indicating heterogeneity and supporting the use of a random effects model. Table 3 presents meta-analytical summary statistics for the association between the five-factor model of personality and gambling for all 20 samples. Figures 2, 3, 4, 5 and 6 illustrate the analyses of each individual personality factor and its association with problem gambling for the 20 samples included in the meta-analysis.

**Table 3:** Summary statistics for the meta-analysis of the association between pathological gambling and the five-factor model of personality

Analysis	*N*	Openness		Conscientiousness		Extraversion		Agreeableness		Neuroticism	
		*r*(95% CI)	*p*	*r*(95% CI)	*p*	*r*(95% CI)	*p*	*r*(95% CI)	*p*	*r*(95% CI)	*p*
Bagby et al., 2007	283	− 0.05(-0.16,0.07)	0.447	− 0.26(-0.36,-0.15)	< 0.001	− 0.10(-0.22,0.01)	0.080	− 0.12(-0.24,-0.01)	0.036	0.24(0.13,0.35)	< 0.001
Brunborg et al., 2016	9111	− 0.01(-0.03,0.01)	0.276	− 0.06(-0.08,-0.04)	< 0.001	− 0.02(-0.04,0.00)	0.137	− 0.05(-0.07,-0.02)	0.000	0.07(0.05,0.09)	< 0.001
Buckle et al., 2013^a^	212	− 0.22(-0.34,-0.08)	0.002	− 0.04(-0.17,0.10)	0.593	0.03(-0.11,0.16)	0.707	− 0.18(-0.31,-0.05)	0.009	0.01(-0.12,0.14)	0.885
Carlotta et al., 2015	110	− 0.35(-0.50,-0.18)	< 0.001	− 0.31(-0.47,-0.14)	0.000	− 0.11(-0.29,0.07)	0.231	− 0.10(-0.28,0.09)	0.302	0.05(-0.14,0.23)	0.615
Cerasa et al., 2018	200	− 0.27(-0.40,-0.14)	< 0.001	− 0.19(-0.32,-0.06)	0.006	− 0.10(-0.24,0.03)	0.140	0.03(-0.11,0.16)	0.714	0.22(0.08,0.35)	0.002
Crossman, 2007	206	− 0.08(-0.21,0.06)	0.269	− 0.27(-0.39,-0.14)	< 0.001	− 0.06(-0.19,0.08)	0.408	− 0.22(-0.35,-0.09)	0.001	0.13(-0.01,0.26)	0.065
Dash et al., 2019 men^b^	1365	− 0.10(-0.15,-0.05)	< 0.001	− 0.61(-0.64,-0.58)	< 0.001	− 0.18(-0.23,-0.13)	< 0.001	− 0.59(-0.62,-0.55)	< 0.001	0.58(0.54,0.61)	< 0.001
Dash et al., 2019 women^b^	2420	− 0.14(-0.18,-0.10)	< 0.001	− 0.62(-0.64,-0.59)	< 0.001	− 0.59 (-0.62,-0.56)	< 0.001	− 0.69(-0.71,-0.67)	< 0.001	0.81(0.80,0.82)	< 0.001
Gong & Zhu, 2019	4100	− 0.01(-0.04,0.02)	0.393	− 0.10(-0.13,-0.07)	< 0.001	0.00(-0.03,0.03)	1.000	− 0.06(-0.09,-0.03)	< 0.001	0.11(0.07,0.14)	< 0.001
Hwang et al., 2012	48	− 0.39(-0.59,-0.14)	0.003	− 0.43(-0.62,-0.19)	< 0.001	− 0.05(-0.32,0.23)	0.742	− 0.07(-0.34,0.21)	0.628	0.18(-0.10,0.43)	0.198
Kaare et al., 2009	69	0.07(-0.17,0.30)	0.569	− 0.29(-0.49,-0.07)	0.011	− 0.05(-0.27,0.19)	0.705	− 0.21(-0.42,0.02)	0.077	0.50(0.32,0.65)	< 0.001
Mann et al., 2017^c^	113	− 0.22(-0.38,-0.04)	0.019	− 0.36(-0.51,-0.20)	< 0.001	− 0.22,(-0.39,-0.04)	0.015	− 0.42(-0.55,-0.26)	< 0.001	0.49(0.35,0.61)	< 0.001
Miller et al., 2013	354	− 0.13(-0.23,-0.03)	0.014	− 0.10(-0.20,0.01)	0.074	− 0.11(-0.21,0.00)	0.048	− 0.13(-0.23,-0.02)	0.019	0.23(0.12,0.32)	< 0.001
Müller et al., 2014	215	− 0.58(-0.66,-0.50)	< 0.001	− 0.17(-0.30,-0.04)	0.009	− 0.09(-0.22,0.04)	0.185	− 0.26(-0.38,-0.13)	0.000	0.29(0.16,0.40)	< 0.001
Myrseth et al., 2009	156	− 0.51(-0.61,-0.39)	< 0.001	− 0.44(-0.55,-0.31)	< 0.001	− 0.17(-0.32,-0.02)	0.027	− 0.17(-0.32,-0.02)	0.031	0.46(0.33,0.57)	< 0.001
Quilty et al., 2021	134	− 0.18(-0.34,-0.01)	0.033	− 0.28(-0.42,-0.12)	0.001	− 0.14(-0.30,0.03)	0.109	− 0.15(-0.31,0.02)	0.076	0.50(0.37,0.61)	< 0.001
Tabri et al., 2017	197	− 0.14(-0.27,0.00)	0.050	− 0.28(-0.41,-0.15)	< 0.001	0.00(-0.14,0.14)	0.989	− 0.20(-0.33,-0.06)	0.006	0.17(0.04,0.31)	0.014
Von der Heiden & Egloff, 2021	12,556	− 0.02(-0.04,0.00)	0.025	− 0.07(-0.09,-0.05)	< 0.001	− 0.01(-0.03,0.01)	0.263	− 0.05(-0.07,-0.03)	< 0.001	0.08(0.06,0.10)	< 0.001
Whiting et al., 2019	248	− 0.09(-0.21,0.03)	0.154	− 0.27(-0.38,-0.15)	< 0.001	− 0.02(-0.14,0.11)	0.765	− 0.23(-0.34,-0.11)	< 0.001	0.39(0.28,0.48)	< 0.001
Zilberman et al., 2018	125	− 0.23(-0.38,-0.06)	0.009	− 0.21(-0.37,-0.04)	0.015	− 0.03(-0.21,0.14)	0.703	− 0.22(-0.38,-0.05)	0.012	0.32(0.16,0.46)	< 0.001

*Note. N* = number of participants in sample

^a^ In the study of Buckle et al. (2013) we used the square root of *r* squared as the effect size

^b^ For the samples of Dash et al. (2019) we used the results for at least 4 symptoms, to match the standards of DSM-5

^c^ We used the sub-sample of non-comorbid pathological gamblers found in Table 3 of the study of Mann et al. (2017)

### Synthesis of results

Table 4 shows the overall effect size for each personality factor, based on a total of 32,222 participants from 20 samples within 19 studies.

**Table 4:** Meta-analysis summary: Random effects model statistics for the association between problem gambling and the five-factor model of personality

Personality factor	*N*	Point estimate (CI 95%)	*p*-value	Heterogeneity analysis			
				*Q*	df	*p*	I-squared
Openness	20	− 0.17(-0.22,-0.12)	< 0.001	248.18	19	< 0.001	92.34
Conscientiousness	20	− 0.28(-0.38,-0.17)	< 0.001	1443.36	19	< 0.001	98.68
Extraversion	20	− 0.11(-0.20,-0.01)	0.02	1011.8	19	< 0.001	98.12
Agreeableness	20	− 0.22(-0.34,-0.10)	< 0.001	1840.20	19	< 0.001	98.97
Neuroticism	20	0.31(0.17,0.44)	< 0.001	2835.68	19	< 0.001	99.33

*Note. N* = number of observed samples

### Publication bias

Funnel plots for all five personality traits show a symmetric distribution. Duval and Tweedie’s (2000) trim and fill method did not suggest trimming any studies. These results suggest an absence of small-study effects. Table 5 shows the classic fail-safe *N* and Orwin’s fail-safe *N* analyses for each personality factor.

**Table 5:** Fail-safe N analyses

Personality factor	*N*	Classic fail-safe N	Orwin’s fail-safe N
Openness	20	957	n/a
Conscientiousness	20	4514	13
Extraversion	20	869	n/a
Agreeableness	20	3471	10
Neuroticism	20	6541	20

*Note. N* = number of observed samples; n/a = not applicable because the small correlation set as the standard for Orwin’s fail-safe (-0.10) exceeds correlation in observed studies

## Discussion

The present meta-analysis provided a statistical review of the association between the five-factor model of personality and problem gambling. The findings from the 20 samples supported the hypothesis that gambling disorder was significantly associated with higher scores on neuroticism, and lower scores on conscientiousness and agreeableness. The results also showed problem gambling was significantly associated with lower scores on both openness and extraversion.

Cohen (1988) suggested that *r* be interpreted as a small effect when *r* = .10, a medium effect when *r* = .30, and a large effect when *r* = .50. The effect size for neuroticism, 0.31, was a medium effect, and the effect size for conscientiousness, − 0.28, was nearly medium. The other effect sizes were small.

Because the findings are correlational, they do not provide evidence that the traits cause problem gambling. However, the findings are consistent with possible causes of problem gambling. The implications of the findings vary from trait to trait, as described below.

Individuals scoring high on neuroticism tend to be worrisome, anxious, self-conscious, and depressed. Hence, some individuals may use gambling to escape these negative feelings, at least for a short while (Mackinnon et al., 2016).

Low conscientiousness involves apathy, impulsivity and a disregard of social norms. Impulsivity could play a factor in problem gambling by its focus on the extreme short-term over the longer term (Ioannidis et al., 2019).

Low agreeableness is characterized by a tendency to be unsocial, inconsiderate, and competitive. Disagreeable behavior may be an antecedent to relationship and occupational dysfunction, consequences that are characteristic of gambling disorder (Widinghoff et al., 2019). The competitive element of this trait could lead individuals to continue gambling despite losses (Parke et al., 2004).

Individuals low in openness tend to avoid change, to be closed-minded, and to prefer routine. The change-avoidant characteristic could contribute to persistent gambling by keeping a person repeating the behavior that is causing problems (Myrseth et al., 2009). The relationship between extraversion and problem gambling was the lowest in magnitude among the five personality factors. Low extraversion involves low engagement with others and is typically associated with maladaptive emotion regulation strategies (Baranczuk, 2019). Low extraversion could help keep some individuals gambling because of a perceived lack of other social sources of excitement, and because of low mood that can be briefly improved by the excitement of gambling.

The meta-analysis showed that all the five-factor personality traits are related to problem gambling in specific ways. However, those same, seemingly undesirable traits might have adaptive value in certain situations. For instance, low agreeableness might help a person avoid being swindled by a new romantic partner.

The personality characteristics associated with problem gambling tend to be associated with other addictive disorders, including alcohol, cannabis, and nicotine use disorders (Dash & Slutske, 2019; Malouff et al., 2005; Müller et al., 2014). Studies have shown that the same personality profile is associated with various psychological problems (Malouff et al., 2005) and with the dramatic and emotional cluster (cluster B) of personality disorders (Samuel & Widiger, 2008; Quilty et al., 2021). It is therefore not surprising that problem gamblers are highly comorbid with nicotine dependence, substance use disorders, mood disorders, anxiety disorders (Lorains et al., 2011), and cluster B personality disorders, particularly borderline personality disorder (Brown et al., 2015). It could be that personality factors help push a person toward addictive behavior.

The main limitations of the present meta-analysis are that the findings (a) are correlational, (b) are entirely based on self-report, (c) combine problems relating to various types of gambling, and (d) are based on mainly English-speaking participants. The correlational findings do not show the direction of the causal relationship between personality and problem gambling. Personality may cause problem gambling, problem gambling may lead to certain personality traits, the relationship may be bidirectional, or some third variable, such as specific genes, may lead to both certain personality traits and problem gambling. Self-report measures rely on a person’s insight and honesty, making them vulnerable to biases. Individuals problematically engaging in different types of gambling activities, e.g., betting on horse races and playing slot machines, may differ in important ways. Individuals who are problem gamblers in different cultures might show a different pattern of personality. It is unknown whether the findings of this meta-analysis could be generalised to every type of gambling and every culture.

The present meta-analysis has advantages over the results of any single study in that the meta-analysis included results from different sets of researchers examining individuals in different countries, used different measures, and analyzed a very large overall group of participants. Aggregating findings across many different studies helps increase the generalizability of findings.

If problem gambling results from attempts to reduce the negative affect of neuroticism, implementing treatment strategies that reduce negative affect may prove helpful in managing problem gambling. Clinicians could devise treatment plans to focus on identifying and implementing ways to improve an individual’s overall affective state. Additionally, clinicians may need to consider the possible influence of personality on treatment processes. The personality profile associated with gambling disorder, including low conscientiousness and low agreeableness, may make it challenging to successfully treat individuals for problem gambling. A person with the personality of low conscientiousness and low agreeableness may not consistently attend appointments or undertake therapeutic assignments. Clinicians may need to make special efforts to overcome these client tendencies. In this regard, Ramos-Grille et al. (2014) found problem gamblers with low scores on conscientiousness had higher rates of treatment failure and relapse.

Clinicians who help problem gamblers could consider personality-focused strategies that have shown success with other addictive disorders; for instance, the Preventure Programme delivers brief interventions targeting personality risk factors associated with substance abuse. The interventions include psychoeducation, motivational enhancement therapy, and cognitive behavioral therapy (Edalati & Conrod, 2019).

Future research on personality and problem gambling could explore whether the findings of the meta-analysis apply to problems with specific types of gambling and apply in cultures not examined so far. Studies could examine whether personality-focused preventive efforts and treatments are effective. Studies could also examine whether different types of treatment for problem gambling change specific personality traits.
